# Supplementary material for: Mapping the evolution of fertility support policies in China: A content and instrumental analysis
Source: PLoS One. 2025 Oct 9;20(10):e0332137. doi: 10.1371/journal.pone.0332137 (PMC12510515; doi:10.1371/journal.pone.0332137)
Supplement: S1 Appendix — (ZIP) [file pone.0332137.s001.zip › S1 Appendix. 226 original policy documents/140-中共中央、国务院印发《成渝地区双城经济圈建设规划纲要》(FBM-CLI-16-5077817).doc]

中共中央、国务院印发《成渝地区双城经济圈建设规划纲要》

**发布部门：** [中国共产党中央委员会](https://www.pkulaw.com/law/chl?Aggs.IssueDepartment=a01) [国务院](https://www.pkulaw.com/law/chl?Aggs.IssueDepartment=5)

**发布日期：**2021.10.20

**实施日期：**2021.10.20

**时效性：** [现行有效](https://www.pkulaw.com/law/chl?Aggs.TimelinessDic=01)

**效力级别：** [党内法规制度](https://www.pkulaw.com/law/chl?Aggs.EffectivenessDic=XR12)

**法规类别：** [区域经济开发](https://www.pkulaw.com/law/chl?Aggs.Category=02308) [营商环境优化](https://dobusiness.pkulaw.com/)

**专题分类：** [长江经济带发展](https://www.pkulaw.com/law/chl?Aggs.SpecialType=022)

中共中央、国务院印发《成渝地区双城经济圈建设规划纲要》

　　近日，中共中央、国务院印发了《成渝地区双城经济圈建设规划纲要》，并发出通知，要求各地区各部门结合实际认真贯彻落实。
　　《成渝地区双城经济圈建设规划纲要》主要内容如下。

目录

　　前言
　　第一章　规划背景
　　第二章　总体要求
　　第一节　指导思想
　　第二节　主要原则
　　第三节　战略定位
　　第四节　发展目标
　　第三章　构建双城经济圈发展新格局
　　第一节　提升双城发展能级
　　第二节　培育发展现代化都市圈
　　第三节　促进双圈互动两翼协同
　　第四节　分类推进大中小城市和县城发展
　　第四章　合力建设现代基础设施网络
　　第一节　构建一体化综合交通运输体系
　　第二节　强化能源保障
　　第三节　加强水利基础设施建设
　　第五章　协同建设现代产业体系
　　第一节　推动制造业高质量发展
　　第二节　大力发展数字经济
　　第三节　培育发展现代服务业
　　第四节　建设现代高效特色农业带
　　第六章　共建具有全国影响力的科技创新中心
　　第一节　建设成渝综合性科学中心
　　第二节　优化创新空间布局
　　第三节　提升协同创新能力
　　第四节　营造鼓励创新的政策环境
　　第七章　打造富有巴蜀特色的国际消费目的地
　　第一节　营造高品质消费空间
　　第二节　构建多元融合的消费业态
　　第三节　塑造安全友好的消费环境
　　第八章　共筑长江上游生态屏障
　　第一节　推动生态共建共保
　　第二节　加强污染跨界协同治理
　　第三节　探索绿色转型发展新路径
　　第九章　联手打造内陆改革开放高地
　　第一节　加快构建对外开放大通道
　　第二节　高水平推进开放平台建设
　　第三节　加强国内区域合作
　　第四节　营造一流营商环境
　　第五节　增强市场主体活力
　　第六节　探索经济区与行政区适度分离改革
　　第十章　共同推动城乡融合发展
　　第一节　推动城乡要素高效配置
　　第二节　推动城乡公共资源均衡配置
　　第三节　推动城乡产业协同发展
　　第十一章　强化公共服务共建共享
　　第一节　推进基本公共服务标准化便利化
　　第二节　共享教育文化体育资源
　　第三节　推动公共卫生和医疗养老合作
　　第四节　健全应急联动机制
　　第十二章　推进规划实施
　　第一节　加强党的集中统一领导
　　第二节　强化组织实施
　　第三节　完善配套政策体系
　　第四节　健全合作机制

前言

　　党中央、国务院高度重视成渝地区发展。2020年1月3日，习近平总书记主持召开中央财经委员会第六次会议，作出推动成渝地区双城经济圈建设、打造高质量发展重要增长极的重大决策部署，为未来一段时期成渝地区发展提供了根本遵循和重要指引。
　　成渝地区双城经济圈位于“一带一路”和长江经济带交汇处，是西部陆海新通道的起点，具有连接西南西北，沟通东亚与东南亚、南亚的独特优势。区域内生态禀赋优良、能源矿产丰富、城镇密布、风物多样，是我国西部人口最密集、产业基础最雄厚、创新能力最强、市场空间最广阔、开放程度最高的区域，在国家发展大局中具有独特而重要的战略地位。为加强顶层设计和统筹协调，加快推动成渝地区形成有实力、有特色的双城经济圈，编制本规划纲要。
　　规划范围包括重庆市的中心城区及万州、涪陵、綦江、大足、黔江、长寿、江津、合川、永川、南川、璧山、铜梁、潼南、荣昌、梁平、丰都、垫江、忠县等27个区（县）以及开州、云阳的部分地区，四川省的成都、自贡、泸州、德阳、绵阳（除平武县、北川县）、遂宁、内江、乐山、南充、眉山、宜宾、广安、达州（除万源市）、雅安（除天全县、宝兴县）、资阳等15个市，总面积18.5万平方公里，2019年常住人口9600万人，地区生产总值近6.3万亿元，分别占全国的1.9%、6.9%、6.3%。
　　本规划纲要是指导当前和今后一个时期成渝地区双城经济圈建设的纲领性文件，是制定相关规划和政策的依据。规划期至2025年，展望到2035年。

　第一章　规划背景

　　“十三五”以来，成渝地区发展驶入快车道。中心城市辐射带动作用持续提升，中小城市加快发展，基础设施更加完备，产业体系日渐完善，科技实力显著增强，内需空间不断拓展，对外交往功能进一步强化。到2019年，地区生产总值年均增长8%以上，社会消费品零售总额年均增长10%以上，常住人口城镇化率超过60%，铁路密度达3.5公里/百平方公里，机场群旅客吞吐量超过1亿人次，常住人口规模、地区经济总量占全国比重持续上升，呈现出重庆和成都双核相向发展、联动引领区域高质量发展的良好态势，已经成为西部地区经济社会发展、生态文明建设、改革创新和对外开放的重要引擎。与此同时，成渝地区综合实力和竞争力仍与东部发达地区存在较大差距，特别是基础设施瓶颈依然明显，城镇规模结构不尽合理，产业链分工协同程度不高，科技创新支撑能力偏弱，城乡发展差距仍然较大，生态环境保护任务艰巨，民生保障还存在不少短板。
　　当今世界正经历百年未有之大变局，新一轮科技革命和产业变革深入发展，国际分工体系面临系统性调整。我国已转向高质量发展阶段，共建“一带一路”、长江经济带发展、西部大开发等重大战略深入实施，供给侧结构性改革稳步推进，扩大内需战略深入实施，为成渝地区新一轮发展赋予了全新优势、创造了重大机遇。在这样的背景下，推动成渝地区双城经济圈建设，符合我国经济高质量发展的客观要求，是新形势下促进区域协调发展，形成优势互补、高质量发展区域经济布局的重大战略支撑，也是构建以国内大循环为主体、国内国际双循环相互促进新发展格局的一项重大举措，有利于在西部形成高质量发展的重要增长极，增强人口和经济承载力；有助于打造内陆开放战略高地和参与国际竞争的新基地，助推形成陆海内外联动、东西双向互济的对外开放新格局；有利于吸收生态功能区人口向城市群集中，使西部形成优势区域重点发展、生态功能区重点保护的新格局，保护长江上游和西部地区生态环境，增强空间治理和保护能力。

　第二章　总体要求

　　加强顶层设计和统筹协调，牢固树立一体化发展理念，唱好“双城记”，共建经济圈，合力打造区域协作的高水平样板，在推进新时代西部大开发中发挥支撑作用，在共建“一带一路”中发挥带动作用，在推进长江经济带绿色发展中发挥示范作用。

　第一节　指导思想

　　以习近平新时代中国特色社会主义思想为指导，全面贯彻党的十九大和十九届二中、三中、四中、五中全会精神，坚持党中央集中统一领导，坚定不移贯彻新发展理念，坚持稳中求进工作总基调，以推动高质量发展为主题，以深化供给侧结构性改革为主线，立足构建以国内大循环为主体、国内国际双循环相互促进的新发展格局，围绕推动形成优势互补、高质量发展的区域经济布局，强化重庆和成都中心城市带动作用，引领带动成渝地区统筹协同发展，促进产业、人口及各类生产要素合理流动和高效集聚，加快形成改革开放新动力，加快塑造创新发展新优势，加快构建与沿海地区协作互动新局面，加快拓展参与国际合作新空间，推动成渝地区形成有实力、有特色的双城经济圈，打造带动全国高质量发展的重要增长极和新的动力源。

　第二节　主要原则

　　--双核引领，区域联动。提升重庆、成都中心城市综合能级和国际竞争力，处理好中心和区域的关系，强化协同辐射带动作用，以大带小、加快培育中小城市，以点带面、推动区域均衡发展，以城带乡、有效促进乡村振兴，形成特色鲜明、布局合理、集约高效的城市群发展格局。
　　--改革开放，创新驱动。充分发挥市场在资源配置中的决定性作用，更好发挥政府作用，强化改革的先导和突破作用，积极推动更高层次开放。强化体制创新，面向国内外集聚创新资源，推动科技创新应用与产业转型升级深度融合，构建协同创新体系。
　　--生态优先，绿色发展。全面践行生态文明理念，强化长江上游生态大保护，严守生态保护红线、永久基本农田、城镇开发边界三条控制线，优化国土空间开发格局，提高用地、用水、用能效率，构建绿色低碳的生产生活方式和建设运营模式，实现可持续发展。
　　--共享包容，改善民生。坚持以人民为中心的发展思想，增加优质公共产品和服务供给，持续改善民生福祉，构建多元包容的社会治理格局，让改革发展成果更多更公平惠及人民，提高人民群众获得感、幸福感、安全感。
　　--统筹协同，合作共建。坚持“川渝一盘棋”思维，发挥优势、错位发展，优化整合区域资源，加强交通、产业、科技、环保、民生政策协同对接，做到统一谋划、一体部署、相互协作、共同实施，辐射带动周边地区发展，显著提升区域整体竞争力。

　第三节　战略定位

　　尊重客观规律，发挥比较优势，把成渝地区双城经济圈建设成为具有全国影响力的重要经济中心、科技创新中心、改革开放新高地、高品质生活宜居地。
　　具有全国影响力的重要经济中心。依托综合交通枢纽和立体开放通道，提高参与全球资源配置能力和整体经济效率，培育竞争优势突出的现代产业体系，发展富有巴蜀特色的多元消费业态，打造西部金融中心、国际消费目的地，共建全国重要的先进制造业基地和现代服务业高地。
　　具有全国影响力的科技创新中心。紧抓新一轮科技革命机遇，发挥科教人才和特色产业优势，推动创新环境优化，加强创新开放合作，促进创新资源集成，激发各类创新主体活力，大力推进科技和经济发展深度融合，打造全国重要的科技创新和协同创新示范区。
　　改革开放新高地。积极推进要素市场化配置、科研体制、跨行政区经济社会管理等重点领域改革。依托南向、西向、东向大通道，扩大全方位高水平开放，形成“一带一路”、长江经济带、西部陆海新通道联动发展的战略性枢纽，成为区域合作和对外开放典范。
　　高品质生活宜居地。大力推进生态文明建设，筑牢长江上游生态屏障，在西部地区生态保护中发挥示范作用，促进社会事业共建共享，大幅改善城乡人居环境，打造世界级休闲旅游胜地和城乡融合发展样板区，建设包容和谐、美丽宜居、充满魅力的高品质城市群。

　第四节　发展目标

　　到2025年，成渝地区双城经济圈经济实力、发展活力、国际影响力大幅提升，一体化发展水平明显提高，区域特色进一步彰显，支撑全国高质量发展的作用显著增强。
　　--双城引领的空间格局初步形成。重庆、成都作为国家中心城市的发展能级显著提升，区域带动力和国际竞争力明显增强。都市圈同城化取得显著突破，中小城市和县城发展提速，大中小城市和小城镇优势互补、分工合理、良性互动、协调发展的城镇格局初步形成，常住人口城镇化率达到66%左右。
　　--基础设施联通水平大幅提升。现代化多层次轨道交通网络初步建成，出渝出川四向通道基本形成，重庆、成都间1小时可达，铁路网总规模达到9000公里以上、覆盖全部20万以上人口城市，航空枢纽地位更加凸显，长江上游航运中心和物流中心基本建成，5G网络实现城镇和重点场景全覆盖，新型基础设施水平明显提高，能源保障能力进一步增强。
　　--现代经济体系初步形成。区域协同创新体系基本建成，研发投入强度达到2.5%左右，科技进步贡献率达到63%，科技创新中心核心功能基本形成。优势产业区域内分工更加合理、协作效率大幅提升，初步形成相对完整的区域产业链供应链体系，呈现世界级先进制造业集群雏形，数字经济蓬勃发展，西部金融中心初步建成，现代服务业优势明显增强。
　　--改革开放成果更加丰硕。制度性交易成本明显降低，跨行政区利益共享和成本共担机制不断创新完善，阻碍生产要素自由流动的行政壁垒和体制机制障碍基本消除，营商环境达到国内一流水平，统一开放的市场体系基本建立。重庆、四川自由贸易试验区等重大开放平台建设取得突破，协同开放水平显著提高，内陆开放战略高地基本建成，对共建“一带一路”支撑作用显著提升。
　　--生态宜居水平大幅提高。生态安全格局基本形成，环境突出问题得到有效治理，生态环境协同监管和区域生态保护补偿机制更加完善，地级及以上城市空气质量优良天数比率达到88%，跨界河流断面水质达标率达到95%，河流主要断面生态流量满足程度达到90%以上，城市开发模式更加集约高效，公共服务便利共享水平明显提高，精细化治理能力显著增强。
　　到2035年，建成实力雄厚、特色鲜明的双城经济圈，重庆、成都进入现代化国际都市行列，大中小城市协同发展的城镇体系更加完善，基础设施互联互通基本实现，具有全国影响力的科技创新中心基本建成，世界级先进制造业集群优势全面形成，现代产业体系趋于成熟，融入全球的开放型经济体系基本建成，人民生活品质大幅提升，对全国高质量发展的支撑带动能力显著增强，成为具有国际影响力的活跃增长极和强劲动力源。

　第三章　构建双城经济圈发展新格局

　　以发挥优势、彰显特色、协同发展为导向，突出双城引领，强化双圈互动，促进两翼协同，统筹大中小城市和小城镇发展，促进形成疏密有致、集约高效的空间格局。

　第一节　提升双城发展能级

　　面向新发展阶段、着眼现代化，优化重庆主城和成都功能布局，全面提升发展能级和综合竞争力，引领带动双城经济圈发展。
　　重庆。以建成高质量发展高品质生活新范例为统领，在全面深化改革和扩大开放中先行先试，建设国际化、绿色化、智能化、人文化现代城市，打造国家重要先进制造业中心、西部金融中心、西部国际综合交通枢纽和国际门户枢纽，增强国家中心城市国际影响力和区域带动力。以长江、嘉陵江为主轴，沿三大平行槽谷组团式发展，高标准建设两江新区、西部（重庆）科学城等，重塑“两江四岸”国际化山水都市风貌。
　　成都。以建成践行新发展理念的公园城市示范区为统领，厚植高品质宜居优势，提升国际国内高端要素运筹能力，构建支撑高质量发展的现代产业体系、创新体系、城市治理体系，打造区域经济中心、科技中心、世界文化名城和国际门户枢纽，提升国家中心城市国际竞争力和区域辐射力。高水平建设天府新区、西部（成都）科学城等，形成“一山连两翼”城市发展新格局。

　第二节　培育发展现代化都市圈

　　把握要素流动和产业分工规律，围绕重庆主城和成都培育现代化都市圈，带动中心城市周边市地和区县加快发展。
　　重庆都市圈。梯次推动重庆中心城区与渝西地区融合发展。畅通璧山、江津、长寿、南川联系中心城区通道，率先实现同城化。强化涪陵对渝东北、渝东南带动功能，支持永川建设现代制造业基地和西部职教基地，支持合川加快发展网络安全产业、推动建成区域性公共服务中心，推进綦江、万盛一体建设西部陆海新通道渝黔综合服务区和渝黔合作先行示范区，打造重庆中心城区辐射带动周边的战略支点。推进重庆向西发展，提升荣昌、铜梁、大足、潼南特色化功能，建设与成都相向发展的桥头堡。推动广安全面融入重庆都市圈，打造川渝合作示范区。
　　成都都市圈。充分发挥成都带动作用和德阳、眉山、资阳比较优势，加快生产力一体化布局，促进基础设施同网、公共服务资源共享、政务事项通办、开放门户共建，创建成德眉资同城化综合试验区，建设经济发达、生态优良、生活幸福的现代化都市圈。推动成都、德阳共建重大装备制造基地，打造成德临港经济产业带。加快天府新区成都片区和眉山片区融合发展，打造成眉高新技术产业带。促进成都空港新城与资阳临空经济区协同发展，打造成资临空经济产业带。推动成都东进，以促进制造业高质量发展为重点将成都东部建成与重庆联动的重要支点。

　第三节　促进双圈互动两翼协同

　　依托资源禀赋、人员往来、产业联系等方面优势，强化区域中心城市互动和毗邻地区协同，优化成渝地区双城经济圈协同发展格局。
　　推动重庆、成都都市圈相向发展。依托成渝北线、中线和南线综合运输通道，夯实成渝主轴发展基础，强化重庆都市圈和成都都市圈互动。支持遂宁与潼南、资阳与大足等探索一体规划、成本共担、利益共享的建设模式。强化都市圈辐射作用，带动成都平原一体化发展，把绵阳、乐山打造为成都平原区域中心城市，支持雅安建设绿色发展示范市，支持黔江建设渝东南区域中心城市。
　　推动渝东北、川东北地区一体化发展。支持万州建设渝东北区域中心城市，支持南充、达州建设川东北区域中心城市，发挥垫江、梁平、丰都、忠县、云阳节点作用，带动双城经济圈北翼发展。支持万州、达州、开州共建川渝统筹发展示范区，加强规划、政策、项目统筹，在产业发展、公共服务、生态环保等领域探索建立符合高质量发展要求的利益共享机制。
　　推动川南、渝西地区融合发展。支持宜宾、泸州建设川南区域中心城市，推动内江、自贡同城化，带动双城经济圈南翼跨越发展。支持自贡、泸州、内江、宜宾、江津、永川、荣昌等共建川南渝西融合发展试验区，探索建立重大政策协同、重点领域协作、市场主体联动机制，协同建设承接产业转移创新发展示范区，打造西部陆海新通道和长江经济带物流枢纽。
　　辐射带动川渝两省市全域发展。强化双城经济圈对重庆市、四川省其他地区特色产业发展、基础设施建设的引领带动，促进基本公共服务均等化，引导秦巴山区、武陵山区、乌蒙山区、涉藏州县、大小凉山等周边欠发达地区人口向双城经济圈集中，强化生态环境保护，切实巩固提升脱贫成果，促进城乡区域协调发展。

　第四节　分类推进大中小城市和县城发展

　　分类指导、科学施策，推动公共资源在双城经济圈各级各类城市间合理配置，优化城市规模结构和功能布局。
　　推动超大特大城市中心城区瘦身健体。统筹兼顾经济、生态、安全、健康等多元需求，推动重庆和成都中心城区功能升级，合理控制规模，优化开发格局，推动城市发展由外延扩张式向内涵提升式转变，防止城市“摊大饼”，积极破解“大城市病”，合理控制开发强度和人口密度。集聚创新要素，增强高端服务功能，率先形成以现代服务业为主体、先进制造业为支撑的产业结构。建设产城融合、职住平衡、生态宜居、交通便利的郊区新城，实现多中心、串联式、组团化发展。
　　加快提升大中城市产业水平和功能品质。主动承接超大特大城市产业转移和功能疏解，夯实实体经济发展基础。立足特色资源和产业基础，推动制造业差异化、规模化、集群化发展，因地制宜打造先进制造业基地、商贸物流中心和区域专业服务中心。优化大中城市管辖范围和市辖区规模结构。支持三级医院和新建高校、高校新建校区在大中城市布局，增加医疗、文化、体育资源供给。优化市政设施功能，改善人居环境，营造现代时尚的消费场景，提升城市生活品质。
　　推进县城城镇化补短板强弱项。加快县城城镇化建设，推动农业转移人口就地就近城镇化。推动重庆市郊区和四川省县城及县级市城区公共服务设施、环境卫生基础设施、市政公用设施、县域经济培育设施提级扩能，推动公共资源适当向县城（郊区、县级市城区）倾斜，补齐短板、补强弱项，提升县域经济发展能力。引导产业项目向资源环境承载力强、发展潜力大的县城（郊区、县级市城区）布局，培育壮大特色优势产业。推动具备条件的县有序改市。
　　分类引导小城镇发展。切实放权赋能，支持位于都市圈范围内的重点镇加强与周边城市的规划统筹、功能配套，分担城市功能。通过规划引导、市场运作，将具有特色资源、区位优势的小城镇培育成为专业特色镇。引导一般小城镇完善基础设施和公共服务，增强服务农村、带动周边功能。

　第四章　合力建设现代基础设施网络

　　以提升内联外通水平为导向，强化门户枢纽功能，加快完善传统和新型基础设施，构建互联互通、管理协同、安全高效的基础设施网络。

　第一节　构建一体化综合交通运输体系

　　打造国际航空门户枢纽。高质量建成成都天府国际机场，打造国际航空枢纽，实施双流国际机场扩能改造，实现天府国际机场与双流国际机场“两场一体”运营。推进重庆江北国际机场改扩建，规划研究重庆新机场建设，提升重庆国际枢纽功能。布局建设乐山、阆中、遂宁、雅安等一批支线机场，研究广安机场建设。织密国际航线网络，提高与全球主要城市之间的通达性。推动两省市机场集团交叉持股，强化城市群机场协同运营，合力打造世界级机场群。优化空域结构，提升空域资源配置使用效率。深化低空空域管理改革，加快通用航空发展。
　　共建轨道上的双城经济圈。科学规划干线铁路、城际铁路、都市圈市域（郊）铁路和城市轨道交通，完善多层次轨道交通网络体系。规划建设川藏铁路，适时推动引入成都枢纽的天府－朝阳湖铁路项目实施。加快建设成都至西宁、重庆至昆明、成都至自贡至宜宾、重庆至黔江、郑州至万州铁路襄阳至万州段等铁路项目，规划建设重庆至万州、成都至达州至万州、重庆至西安、重庆至宜昌、成渝中线等铁路项目，规划研究重庆至贵阳铁路，研究论证重庆至自贡至雅安铁路，拓展出渝出川客运大通道。推进叙永至毕节等铁路及铁路专用线等货运设施建设，逐步恢复沪汉蓉铁路货运功能，完善货运通道布局。研究规划重庆都市圈环线、成都外环、绵遂内等连接重庆中心城区、成都与周边城市的城际铁路和都市圈市域（郊）铁路，优先利用铁路资源开行城际、市域（郊）列车，基本建成中心城市间、中心城市与周边城市（镇）间1小时交通圈和通勤圈。有序推进重庆、成都城市轨道交通规划建设。
　　完善双城经济圈公路体系。畅通对外高速公路通道，强化主要城市间快速联通，加快推进省际待贯通路段建设。提高既有路网通行能力，全面推动G318川藏公路升级改造，加快成渝、渝遂、渝泸、渝邻和成自泸赤等国家高速公路繁忙路段扩能改造，加强干线公路与城市道路有效衔接。优化城际快速路网，疏通主要节点城市进出通道，增强公路对客货运枢纽的集疏运服务能力，提升路网通达效率和安全水平。推动毗邻地区互联互通，建设重庆至合江至叙永、泸州至永川、大足至内江、铜梁至安岳、南充至潼南、大竹至垫江、开江至梁平等高速公路。
　　推动长江上游航运枢纽建设。健全以长江干线为主通道、重要支流为骨架的航道网络，优化干支流水库群联合调度，研究优化长江上游分段通航标准，加快长江上游航道整治和梯级渠化，全面畅通岷江、嘉陵江、乌江、渠江等。推进利泽、白马、犍为、龙溪口、风洞子等航电枢纽建设。加强港口分工协作，构建结构合理、功能完善的港口群，打造要素集聚、功能完善的港航服务体系。组建长江上游港口联盟，加强与上海国际航运中心合作，推进港口企业加强合资合作，促进区域港口码头管理运营一体化。
　　提升客货运输服务水平。推动多层次轨道交通网络运营管理衔接融合，研究建立一体化建设运营机制，推广交通“一卡通”服务和二维码“一码畅行”，加快实现运营公交化。加强机场与轨道交通衔接，完善机场集疏运体系，探索空铁联程联运新技术新模式。推进一体化综合客运枢纽和衔接高效的综合货运枢纽建设，提升枢纽运营智能化水平。推进铁路专用线进重要枢纽型港区、大型工矿企业和物流园区，加快发展铁水、公铁联运和“一单制”联运服务。支持高铁快运、电商快递班列、多式联运班列发展。

　第二节　强化能源保障

　　优化区域电力供给。稳步推进金沙江、雅砻江、大渡河水电基地开发，优先建设具有调节能力的水库电站。统筹推进风光水多能互补能源基地建设，积极推广分布式能源发展，研究开展氢能运营试点示范，建设优质清洁能源基地。优化川渝电力资源配置，完善川渝电网主网架结构，优化重庆都市圈500千伏目标网架。研究论证疆电入渝工程。推进白鹤滩水电站留存部分电量在川渝电网消纳。培育发展电力现货市场和川渝一体化电力辅助服务市场。
　　统筹油气资源开发。发挥长宁－威远、涪陵国家级页岩气示范区引领作用，推动页岩气滚动开发，建设天然气千亿立方米产能基地，打造中国“气大庆”。完善天然气管网布局。优化完善成品油储运设施，有序开展中航油西南战略储运基地、陕西入川渝成品油管道、沿江成品油管道等前期工作。发挥重庆石油天然气交易中心作用，形成具有影响力的价格基准。完善页岩气开发利益共享机制，有序放开油气勘探开发市场，加大安岳等地天然气勘探开发力度。

　第三节　加强水利基础设施建设

　　研究推进跨区域重大蓄水、提水、调水工程建设，增强跨区域水资源调配能力，推动形成多源互补、引排得当的水网体系。推动大型水库及引水供水重点工程建设。有序推进引大济岷、涪江右岸、向家坝灌区二期、长征渠、渝南及重庆中部水资源配置、沱江团结等引水供水重大工程的研究论证。加强大中型灌区续建配套和现代化改造。加强饮用水水源地和备用水源建设，推进人口分散区域重点小型标准化供水设施建设，保障区域供水安全。推进防洪减灾设施建设，加强主要江河和中小河流防洪治理，实施防洪控制性水库联合调度。系统推进城市堤防、排水管渠、排涝除险、蓄水空间等设施建设，有效治理城市内涝问题。构建智慧水利平台，健全水资源监控体系，推进水利资源共享、调配、监管一体化。

　第五章　协同建设现代产业体系

　　以全球新一轮科技革命和产业链重塑为契机，坚持市场主导、政府引导，强化机制创新，优化、稳定、提升产业链供应链，加快构建高效分工、错位发展、有序竞争、相互融合的现代产业体系。

　第一节　推动制造业高质量发展

　　优化重大生产力布局。整合提升优势产业，加快补齐关键短板，增强全产业链优势，形成特色鲜明、相对完整、安全可靠的区域产业链供应链体系。提升重庆、成都产业创新发展能力，打造制造业高质量发展双引擎，推动都市圈外围地区加快发展电子信息、汽车等产业，形成研发在中心、制造在周边、链式配套、梯度布局的都市圈产业分工体系。强化双城经济圈北翼地区先进材料、汽摩配件等产业协作，南翼地区联动集聚食品饮料、装备制造、能源化工、节能环保等产业。
　　培育具有国际竞争力的先进制造业集群。以智能网联和新能源为主攻方向，共建高水平汽车产业研发生产制造基地。聚焦航空航天、轨道交通、能源装备、工业机器人、仪器仪表、数控机床、摩托车等领域，培育世界级装备制造产业集群。整合白酒主产区优质资源，壮大健康食品、精品服饰、特色轻工等产业，培育特色消费品产业集群。深入推进国家战略性新兴产业集群发展工程，前瞻布局一批先导产业，壮大先进材料产业，协同发展生物医药、医疗器械、现代中药产业，共建西部大健康产业基地。
　　大力承接产业转移。发挥要素成本、市场和通道优势，以更大力度、更高标准承接东部地区和境外产业链整体转移、关联产业协同转移，补齐建强产业链。积极发挥产业转移项目库作用，建立跨区域承接产业转移协调机制，完善信息对接、权益分享、税收分成等政策体系。布局产业转移集中承接地，继续安排中央预算内投资支持国家级新区、承接产业转移示范区重点园区的基础设施和公共服务平台建设，不断提升承接产业能力。研究以市场化方式设立区域产业协同发展投资基金，支持先导型、牵引性重大产业项目落地。
　　整合优化重大产业平台。发挥重庆两江新区、四川天府新区旗舰作用，加快重庆经济技术开发区、海峡两岸产业合作区、成都国际铁路港经济开发区及其他国家级、省级开发区建设，推动成都天府临空经济区建设。鼓励涪陵、綦江、合川、资阳、遂宁、宜宾等创建国家高新技术产业开发区，打造一批国家新型工业化产业示范基地，推动建设广安－渝北等一批跨省市毗邻地区产业合作园区。支持自贡等老工业城市转型升级，建设新时代深化改革扩大开放示范城市。创新“一区多园”、“飞地经济”等建园方式，推动各类开发区和产业集聚区政策叠加、服务体系共建。

　第二节　大力发展数字经济

　　布局完善新一代信息基础设施。加快5G网络建设，推进千兆光纤接入网络广泛覆盖，加快推进基于IPv6的下一代互联网部署，推动国家级互联网骨干直联点宽带扩容。统筹布局大型云计算和边缘计算数据中心。完善工业互联网标识解析国家顶级节点功能，加快建设二级节点。积极发展物联网，建设全面覆盖、泛在互联的城市智能感知网络。开展新一代移动通信网络试验验证，实施车联网试点示范建设工程。加快提升传统基础设施智能化水平。
　　合力打造数字产业新高地。聚焦集成电路、新型显示、智能终端等领域，打造“云联数算用”要素集群和“芯屏器核网”全产业链，培育超高清视频、人工智能、区块链、数字文创等创新应用，联手打造具有国际竞争力的电子信息产业集群。大力发展数字经济，推动数字产业化、产业数字化，促进软件、互联网、大数据等信息技术与实体经济深度融合，加快重点领域数字化发展，引领产业转型升级。围绕产业发展需要，推动共建成渝工业互联网一体化发展示范区、区域协同公共服务平台和服务体系，构建全国领先的“5G＋工业互联网”生态。支持联合建设国家数字经济创新发展试验区和国家数字服务出口基地，建设“智造重镇”和“智慧名城”。
　　积极拓展数字化应用。探索建立统一标准、开放互通的公共应用平台，推动双城经济圈政务数据资源共享共用，推动地级以上城市全面建立数字化管理平台。推进城市基础设施、建筑楼宇等的数字化管理，稳步推进“数字＋”与城市运营管理各领域深度融合。完善大数据辅助科学决策机制，加快提高治理数字化水平。适应数字技术全面融入社会交往和日常生活新趋势，促进公共服务、社会运行和治理方式创新，构筑全民畅享的数字生活。
　　全面提升数字安全水平。加强通信网络、重要信息系统和数据资源保护，增强关键信息基础设施安全防护能力。深化网络安全等级保护制度和关键信息基础设施安全保护制度。完善重庆和成都重要数据灾备中心功能，建设联合异地灾备数据基地。建设网络安全产业基地，支持开展法定数字货币研究及移动支付创新应用。

　第三节　培育发展现代服务业

　　推动先进制造业和服务业融合发展。引导制造企业延伸服务链条、发展服务环节，推动生产服务型企业创新服务供给，提升制造业服务化水平和全产业链价值。在研发设计、科技服务、商务咨询、人力资源服务等领域，联合打造一批服务品牌。依托优势企业培育发展工业设计中心，支持食品药品检测基地、重庆工业设计产业城等建设。支持在成渝地区建设国家检验检测高技术服务业集聚区。支持川渝毗邻地区建立人力资源服务产业园。鼓励重庆、成都等开展先进制造业和现代服务业融合发展试点。
　　提升商贸物流发展水平。强化重庆、成都国家物流枢纽功能，合力建设国际货运中心。支持万州、涪陵、长寿、遂宁、达州、泸州、自贡等打造区域性物流中心。支持全货运航空公司在成渝地区设立基地，加快完善多式联运国际物流服务网络，打造多元化、国际化、高水平物流产业体系，培育发展龙头企业。围绕优势产业和主导产品，差异化建设一批内外贸相结合的专业市场。扎实推进跨境电子商务综合试验区建设。大力发展数字商务，探索建立反向定制（C2M）产业基地。强化会展经济对商贸物流的带动作用，联合打造一批专业会展品牌。
　　共建西部金融中心。支持重庆开展区域性股权市场制度和业务创新。支持开展共建“一带一路”金融服务。开展本外币合一账户试点。支持跨境人民币业务创新，探索开展跨国企业集团本外币合一跨境资金池等试点业务，支持在自由贸易试验区设立人民币海外投贷基金。支持开展合格境内投资企业（QDIE）和合格境内有限合伙人（QDLP）试点。积极支持区域金融改革创新，开展绿色金融、金融科技等创新试点，在成都建设基于区块链技术的知识产权融资服务平台。推进金融市场和监管区域一体化，推动在担保、不良资产处置、创业投资和私募股权投资等领域跨区域合作。支持设立市场化征信机构，研发适合西部地区的征信产品，支持中外信用评级机构在成渝地区设立实体机构，推动信用融资产品和服务创新。设立破产法庭，健全金融审判体系。

　第四节　建设现代高效特色农业带

　　推动农业高质量发展。支持川渝平坝和浅丘地区建设国家优质粮油保障基地，打造国家重要的生猪生产基地、渝遂绵优质蔬菜生产带、优质道地中药材产业带、长江上游柑橘产业带和安岳、潼南柠檬产区。推进特色农产品精深加工，打造全球泡（榨）菜出口基地、川菜产业和竹产业基地。发展都市农业，高质量打造成渝都市现代高效特色农业示范区。
　　强化农业科技支撑。共建国家农业高新技术产业示范区。支持建设西南特色作物种质资源库、西部农业人工智能技术创新中心、国家现代农业产业科技创新中心等。推动畜禽遗传资源保护利用，建设区域性畜禽基因库、畜牧科技城、国家级重庆（荣昌）生猪大数据中心。
　　大力拓展农产品市场。积极开展有机产品认证，健全农产品质量安全追溯体系。做强地理标志农产品，推广巴味渝珍、天府龙芽等特色品牌，打造川菜渝味等区域公用品牌。强化农产品分拣、加工、包装、预冷等一体化集配设施建设，大力建设自贡等国家骨干冷链物流基地。大力发展农村电商，建设一批重点网货生产基地和产地直播基地。建设国际农产品加工产业园。

　第六章　共建具有全国影响力的科技创新中心

　　坚定实施创新驱动发展战略，瞄准突破共性关键技术尤其是“卡脖子”技术，强化战略科技力量，深化新一轮全面创新改革试验，增强协同创新发展能力，增进与“一带一路”沿线国家等创新合作，合力打造科技创新高地，为构建现代产业体系提供科技支撑。

　第一节　建设成渝综合性科学中心

　　聚焦核能、航空航天、智能制造和电子信息等领域的战略性产品开发，在四川天府新区、重庆高新区集中布局建设若干重大科技基础设施和一批科教基础设施，引导地方、科研机构和企业建设系列交叉研究平台和科技创新基地，打造学科内涵关联、空间分布集聚的原始创新集群。发挥基础研究和原始创新的引领作用，吸引高水平大学、科研机构和创新型企业入驻，强化开放共享，促进科技成果转化，有效支撑成渝全域高水平创新活动。

　第二节　优化创新空间布局

　　统筹天府国际生物城、未来科技城和成都高新区等资源，建设西部（成都）科学城。瞄准新兴产业设立开放式、国际化高端研发机构，建设重庆两江协同创新区。依托重庆大学城、重庆高新区等，夯实智能产业、生物医学发展基础，建设西部（重庆）科学城。高水平建设中国（绵阳）科技城，鼓励大院大所发展孵化器、产业园。以“一城多园”模式合作共建西部科学城。推动中国科学院等在双城经济圈布局科研平台。

　第三节　提升协同创新能力

　　强化创新链产业链协同。坚持企业主体、市场导向，健全产学研用深度融合的科技创新体系，建设产业创新高地。鼓励有条件的企业组建面向行业共性基础技术、前沿引领技术开发的研究院，支持创新型领军企业联合行业上下游组建创新联合体。支持高校、科研机构和企业共建联合实验室或新型研究机构，共同承担科技项目、共享科技成果。建设一批产业创新中心、技术创新中心、制造业创新中心、工程研究中心等创新平台和综合性检验检测平台。
　　推动区域协同创新。实施成渝科技创新合作计划，联合开展技术攻关，参与实施高分卫星、载人航天、大型飞机、长江上游生态环境修复等国家重大科技任务，积极申报航空发动机、网络空间安全等科技创新重大项目。鼓励共用科技创新平台和大型科研仪器设备，共建创业孵化、科技金融、成果转化平台，打造成渝地区一体化技术交易市场。完善区域知识产权快速协同保护机制，支持设立知识产权法庭。鼓励区域内高校、科研院所、企业共同参与国际大科学计划和大科学工程。

　第四节　营造鼓励创新的政策环境

　　大力吸引创新人才。实施有吸引力的人才政策，引进和培养高水平创新人才队伍，鼓励科技人才在区域内自主流动、择业创业。支持在人才评价、外籍人才引进等政策创新方面先行先试。鼓励成渝地区大学面向全球招生，引进优秀博士后和青年学者。支持引进国内外顶尖高校和科研机构在成渝地区合作建设研究院和研发中心，设立长期、灵活、有吸引力的科研岗位。
　　深化科技创新体制改革。深入推进职务科技成果所有权或长期使用权改革试点，探索高校和科研院所职务科技成果国有资产管理新模式。深化政府部门和科研单位项目资金管理制度改革，允许科研资金跨省市使用。探索建立两省市改革举措和支持政策异地同享机制。
　　健全创新激励政策体系。加大对引进高水平研发机构和先进科技成果的支持力度。综合运用财政、金融等政策手段激励企业加大研发投入力度，引导创业投资机构投资早中期、初创期科技型企业，依法运用技术、能耗、环保等方面的标准促进企业技术改造和新技术应用。支持通过股权与债权相结合等方式，为企业创新活动提供融资服务。支持符合条件的创新型企业上市融资。

　第七章　打造富有巴蜀特色的国际消费目的地

　　以高质量供给引领和创造市场新需求，坚持高端化与大众化并重、快节奏与慢生活兼具，激发市场消费活力，不断增强巴蜀消费知名度、美誉度、影响力。

　第一节　营造高品质消费空间

　　打造城市消费品牌。支持重庆、成都塑造城市特色消费品牌，打造国际消费中心城市。推动涪陵、合川、乐山、雅安、南充等发展人文休闲、度假康养，打造成渝“后花园”。发挥宜宾、泸州白酒品牌优势。推动万州、江津、铜梁、自贡、内江等围绕特色美食、传统工艺产品、民俗节庆、自然遗迹等，建设特色消费聚集区。改造提升商业街区，集聚高端消费资源，打造世界知名商圈。建设一批人文气息浓厚的特色商业名镇。
　　共建巴蜀文化旅游走廊。充分挖掘文化旅游资源，以文促旅、以旅彰文，讲好巴蜀故事，打造国际范、中国味、巴蜀韵的世界级休闲旅游胜地。打造贯通四川、重庆的文化遗产探秘、自然生态体验、红色文化体验等一批精品旅游线路，扩大长江三峡、九寨沟、武隆喀斯特、都江堰－青城山、峨眉山－乐山大佛、三星堆－金沙、三国文化、大足石刻、自贡彩灯等国际旅游品牌影响力。规划建设长征国家文化公园（重庆段、四川段）。打造绵竹熊猫谷和玫瑰谷，探索川西林盘、巴渝村寨保护性开发，依托特色自然风光、民俗风情、农事活动等，发展巴蜀乡村旅游。推动黔江与周边区县文旅融合发展，建设文化产业和旅游产业融合发展示范区。

　第二节　构建多元融合的消费业态

　　推动消费供给升级。促进经典川菜、重庆火锅、盖碗茶等餐饮产品品牌化，创建美食地标。推动传统文化和全新科技元素融入创意设计产业，提升传媒影视、动漫游戏、音乐演艺等产业发展水平，支持举办有国际影响力的时装周、电影节、艺术节等文化展演活动。发展水上运动、山地户外运动、汽车摩托车运动、航空运动等，布局建设自驾游营地和野外露营地，发展乡村民宿，推出温泉、游轮、徒步、自驾等一批特色化、品质化旅游产品，大力发展“旅游＋”产品。提升健康、养老、托育、家政服务等市场化供给质量，壮大社会服务消费。
　　发展消费新场景。引导网络直播、短视频等新消费形态健康发展，促进直播电商、社交电商等线上新模式新业态发展，推动教育、医疗等服务线上线下交互融合。鼓励发展智慧门店、自助终端、智能机器人等“无接触”零售。发展更多参与式、体验式消费模式和业态。发展假日经济，丰富夜市、夜展、夜秀、夜游等夜间经济产品，建设一批夜间文旅消费集聚区，擦亮“两江游”、“街巷游”等夜间经济名片，展现国际时尚范、巴蜀慢生活。

　第三节　塑造安全友好的消费环境

　　完善消费促进政策。规范发展消费金融，在风险可控、商业可持续前提下稳妥开发适应新消费趋势的金融产品和服务。拓展移动支付使用范围，提升境外人员在境内使用移动支付便利化水平。研究将闲置厂房、办公用房等改为商业用途的支持政策。支持符合条件的地区建设市内免税店、口岸免税店、离境提货点。优化离境退税服务，促进国际消费便利化。
　　健全消费者权益保障制度。推动服务标准化建设，发布行业优质企业名录，鼓励企业开展消费体验评价并公开评价结果。对涉及安全、健康、环保等方面的产品依法实施强制性产品认证（CCC认证），建设针对食品、药品等重点产品的溯源公共服务平台，推动溯源信息资源稳妥有序向社会开放。加强重点领域广告监管。鼓励线下实体店自主承诺无理由退货，探索建立特色旅游商品无理由退货制度。健全消费领域信用监管体系，加强信用信息采集，开展消费投诉信息公示，强化社会监督。完善消费环节经营者首问责任制和赔偿先付制度。

　第八章　共筑长江上游生态屏障

　　坚持共抓大保护、不搞大开发，把修复长江生态环境摆在压倒性位置，深入践行绿水青山就是金山银山理念，坚持山水林田湖草是一个生命共同体，深入实施主体功能区战略，全面加快生态文明建设，建立健全国土空间规划体系，形成人与自然和谐共生的格局。

　第一节　推动生态共建共保

　　共建生态网络。构建以长江、嘉陵江、乌江、岷江、沱江、涪江为主体，其他支流、湖泊、水库、渠系为支撑的绿色生态廊道。依托龙门山、华蓥山、大巴山、明月山等，实施森林生态系统休养生息和矿区恢复治理，共筑绿色生态屏障。加大对重点流域、三峡库区“共抓大保护”项目支持力度，实施“两岸青山·千里林带”等生态治理工程。推动大熊猫国家公园建设，加强珍稀濒危动植物保护。加快各类自然保护地整合优化，强化重要生态空间保护。打造龙泉山城市森林公园。强化周边地区生态系统保护和治理，加强三峡库区小流域和坡耕地水土流失综合治理，实施三峡库区消落带治理和岩溶地区石漠化综合治理。
　　共抓生态管控。统筹建立并实施双城经济圈及其周边地区“三线一单”生态环境分区管控制度。加强流域水资源统一管理和联合调度。建立跨流域跨区域横向生态保护补偿机制。加大国家和省级生态保护补偿资金对长江上游生态屏障建设支持力度。严格执行生态损害赔偿制度。试点推进生态敏感区生态搬迁。落实好长江十年禁渔，实施长江上游流域重点水域全面禁捕，严厉打击非法捕捞，建立禁捕长效机制。依法联合查处交界区域破坏生态环境的违法行为。

　第二节　加强污染跨界协同治理

　　统一环保标准。制定统一的环保标准编制技术规范，联合开展现行环保标准差异分析评估，有序制定修订统一的大气、水、土壤以及危险废物、噪声等领域环保标准。坚持一张负面清单管川渝两地，严格执行长江经济带发展负面清单管理制度体系，建立健全生态环境硬约束机制。开展跨区域联合环境执法，统一管控对象的界定标准和管控尺度，共同预防和处置突发环境事件。完善重大基础设施建设项目环境影响评价制度。
　　推进跨界水体环境治理。完善跨省市水体监测网络，建立上下游水质信息共享和异常响应机制。开展联合巡河，加强工业污染、畜禽养殖、入河排污口、环境风险隐患点等协同管理。加强三峡库区入库水污染联合防治，加快长江入河排污口整改提升，统筹规划建设港口船舶污染物接收、转运及处置设施，推进水域“清漂”联动。推动毗邻地区污水处理设施共建共享。支持在长江、嘉陵江一级支流开展水环境治理试点示范，深化沱江、龙溪河、岷江流域水环境综合治理与可持续发展试点。完善饮用水水源地风险联合防控体系。
　　深化大气污染联防联控。建设跨省市空气质量信息交换平台，发挥西南区域空气质量预测预报中心作用，实施联合预报预警。建立重污染天气共同应对机制，推进应急响应一体联动。探索实施细颗粒物（PM2.5）和臭氧（O3）污染连片整治。实施“散乱污”企业清理整治，依法淘汰落后产能，加快淘汰老旧车辆，加强油品质量联合监督。创建清洁能源高质量发展示范区，提高清洁能源消费比例。
　　加强土壤污染及固废危废协同治理。以沿江工业园区、矿山、受污染耕地、污染地块为重点开展修复与治理。推动固体废物区域转移合作，建立健全固体废物信息化监管体系。统筹规划建设工业固体废物资源回收基地和危险废物资源处置中心，加强尾矿库污染治理，推进毗邻地区处置设施共建共享。依法严厉打击危险废物非法跨界转移、倾倒等违法行为。推动地级以上城市医疗废物集中处置设施全覆盖，县级以上城市及县城医疗废物全收集、全处理，并逐步覆盖到建制镇。协同开展“无废城市”建设。

　第三节　探索绿色转型发展新路径

　　构建绿色产业体系。培育壮大节能环保、清洁生产、清洁能源产业，打造国家绿色产业示范基地。联合打造绿色技术创新中心和绿色工程研究中心，实施重大绿色技术研发与示范工程。实施政府绿色采购，推行绿色产品优先。鼓励国家绿色发展基金加大向双城经济圈投资力度。推行企业循环式生产、产业循环式组合、园区循环化改造，开展工业园区清洁生产试点。落实最严格的水资源管理制度，实施节水行动，加大节能技术、节能产品推广应用力度。深化跨省市排污权、水权、用能权、碳排放权等交易合作。
　　倡导绿色生活方式。共建绿色城市标准化技术支撑平台，完善统一的绿色建筑标准及认证体系，推广装配式建筑、钢结构建筑和新型建材。推动可再生能源利用，支持能源互联网创新，统筹布局电动汽车充换电配套设施。加快推进垃圾分类，共建区域一体化垃圾分类回收网络体系。完善对汽车等的强制报废配套政策，统筹布局再生资源分拣中心，建设城市废弃资源循环利用基地。鼓励创建国家生态文明建设示范市县，深入开展爱国卫生运动。
　　开展绿色发展试验示范。支持万州及渝东北地区探索三峡绿色发展新模式，在生态产品价值实现、生态保护和补偿、绿色金融等领域先行先试、尽快突破，引导人口和产业向城镇化地区集聚，走出整体保护与局部开发平衡互促新路径，保护好三峡库区和长江母亲河。支持四川天府新区在公园城市建设中先行先试，开展可持续发展创新示范，实施城市生态用地改革创新，探索建立公园城市规划导则、指标评价、价值转化等体系。支持重庆广阳岛开展长江经济带绿色发展示范。建设沱江绿色发展经济带。

　第九章　联手打造内陆改革开放高地

　　以共建“一带一路”为引领，打造陆海互济、四向拓展、综合立体的国际大通道，加快建设内陆开放枢纽，深入推进制度型开放，聚焦要素市场化配置等关键领域，深化综合配套改革试验，全面提升市场活力，在西部改革开放中发挥示范带动作用。

　第一节　加快构建对外开放大通道

　　合力建设西部陆海新通道。深化西部省区市协作，支持发挥重庆通道物流和运营组织中心、成都国家重要商贸物流中心作用，共同建设跨区域平台，统筹设置境内外枢纽和集货分拨节点。支持建立铁路运输市场化与政府购买服务相结合的定价机制，降低综合运价水平。对接21世纪海上丝绸之路，推动国际陆海贸易新通道合作，与新加坡合作推动东盟及相关国家共同参与通道建设，探讨衔接中国－中南半岛、孟中印缅等经济走廊和中欧班列建设合作。
　　统筹完善亚欧通道。加强协调联动，推动中欧班列高质量发展，打造西向开放前沿高地，紧密对接丝绸之路经济带。统筹优化中欧班列（成渝）去回程线路和运力，推动集结点、代理、运输、仓储、信息等资源共建共享，强化多式联运衔接，探索国际贸易新规则，提高通关便利化水平，增强国际竞争力。完善跨境邮递体系，建设铁路口岸国际邮件互换中心。打造重庆兴隆场、成都北中欧班列枢纽节点。开拓中欧班列中、东通道，积极衔接中蒙俄经济走廊。
　　优化畅通东向开放通道。依托长江黄金水道和沿江铁路，构建通江达海、首尾联动的东向国际开放通道，扩大与日韩、欧美等国家和地区经贸合作。加强陆水、港航联动，开通往返主要港口的“水上穿梭巴士”和铁水联运班列，建设统一运营品牌，提高进出口货物运输效率、降低运输成本。推进沿江省市港口、口岸合作，优化“沪渝直达快线”运行机制，提高通关效率。

　第二节　高水平推进开放平台建设

　　建设川渝自由贸易试验区协同开放示范区。加大力度推进首创性、差异化改革，支持重庆、四川自由贸易试验区协同开放，试行有利于促进跨境贸易便利化的外汇管理政策。探索更加便利的贸易监管制度。在双城经济圈复制推广自由贸易试验区改革创新成果。扩大金融、科技、医疗、贸易和数字经济等领域开放。
　　打造内陆开放门户。以重庆两江新区、四川天府新区为重点，优先布局国家重大战略项目、试点示范项目，创建内陆开放型经济试验区。扩大包括第五航权在内的国际航权开放，按规定积极扩大铁路、港口、机场以适当方式对外开放，合理规划发展综合保税区、保税物流中心（B型）。加快建设中德、中法、中瑞（士）、中意等双边合作园区。培育进口贸易促进创新示范区，建设“一带一路”进出口商品集散中心。
　　高标准实施高层级开放合作项目。推进中新（重庆）战略性互联互通示范项目，合规有序发展供应链金融和特色跨境金融服务平台，依托贸易金融区块链平台，探索形成贸易金融区块链标准体系。推动建设中新金融科技、航空产业、跨境交易、多式联运等领域合作示范区，建设第三方飞机维修中心，共同打造国际数据港。开展中日（成都）城市建设和现代服务业开放合作示范项目，建设药物供应链服务中心、先进医疗服务中心，推动科技、金融等领域合作。
　　共建“一带一路”对外交往中心。支持举办重要国际会议和赛事。支持共建“一带一路”科技创新合作区和国际技术转移中心，共同举办“一带一路”科技交流大会。高标准举办中国国际智能产业博览会、中国西部国际投资贸易洽谈会、中国西部国际博览会、中国（绵阳）科技城国际科技博览会等国际大型会展。深化文化、教育、医疗、体育等领域国际交流，高质量建设国家文化出口基地，支持川剧、彩灯等中国文化走出去。加强国际友好城市往来。支持建立境外专业人才执业制度，放宽境外人员参加各类职业资格（不包括医疗卫生人员资格）考试限制，支持为外籍高层次人才来华投资就业提供入出境和停居留便利。

　第三节　加强国内区域合作

　　加强与西部地区协调联动。加强与关中平原、兰州－西宁城市群联动，深化能源、物流、产业等领域合作，辐射带动西北地区发展。加强与北部湾、滇中城市群协作，把出境出海通道优势转化为贸易和产业优势，促进西南地区全方位开放。深化与黔中城市群合作，带动黔北地区发展。
　　有力支撑长江经济带发展。加强与长江中游和下游协作，共同推动长江经济带绿色发展。促进生态环境联防联治，加快建立长江流域常态化横向生态保护补偿机制。共同推进长江黄金水道、沿江铁路、成品油输送管道等建设。优化沿江经济布局，有序承接产业转移和人口迁移。
　　深化与东部沿海地区交流互动。对接京津冀协同发展、粤港澳大湾区建设、长三角一体化发展等重大战略，加强科技创新合作与科技联合攻关。鼓励与东部沿海城市建立产业合作结对关系，共建跨区域产业园区，促进项目、技术、人才等高效配置。支持沿海港口在双城经济圈设立无水港。深化三峡库区对口支援工作。

　第四节　营造一流营商环境

　　建设高标准市场体系。共建统一的市场规则、互联互通的市场基础设施，加快清理废除妨碍统一市场和公平竞争的各种规定和做法。打破行政区划对要素流动的不合理限制，推动要素市场一体化，探索以电子营业执照为基础，加快建立公共资源交易平台市场主体信息共享与互认机制。探索建立“市场准入异地同标”机制，推进双城经济圈内同一事项无差别受理、同标准办理。全面实施外商投资准入前国民待遇加负面清单管理制度。推动信用一体化建设，逐步形成统一的区域信用政策法规制度和标准体系，支持共同开发适应经济社会发展需求的信用产品。
　　持续转变政府职能。深化“放管服”改革，对标国际一流水平，加快服务型政府建设，最大限度精简行政审批事项和环节，推行政务服务“最多跑一次”改革，发布“零跑腿”事项清单，加快实现区域内“一网通办”。推行企业简易注销登记，开展企业投资项目承诺制改革，深化工程建设项目审批制度改革。优化综合监管体系，建立健全行政执法联动响应和协作机制。

　第五节　增强市场主体活力

　　深化国有企业改革。有力有序推进国有企业混合所有制改革，推动国有企业建立健全有效制衡的现代企业治理体制、高度市场化和灵活高效的现代企业经营机制、激励和约束并重的现代企业激励机制，推进国有企业经理层成员任期制和契约化管理，完善中国特色现代企业制度，切实激发国有企业发展活力和内生动力。支持开展区域性国资国企综合改革试验。支持以市场化方式设立成渝混合所有制改革产业基金，吸引更多资本参与国有企业改革。加快完善国资监管体制，充分发挥国有资本投资运营公司作用，推动国资监管从管企业向管资本转变，真正实现政企分开、政资分开，使国有企业真正成为独立市场主体。深化效率导向的国资经营评价制度改革。
　　大力发展民营经济。建立规范化、常态化政商沟通机制，畅通民营企业反映问题和诉求的渠道。积极缓解民营企业和中小微企业发展难题，创建民营经济示范城市。搭建川商渝商综合服务平台，引导川渝两省市商（协）会和重点民营企业共同开展项目推介、银企对接，鼓励川商渝商回乡创业。支持举办川渝民营经济发展合作峰会。

　第六节　探索经济区与行政区适度分离改革

　　支持在重庆都市圈、成都都市圈以及川渝统筹发展示范区、川南渝西融合发展试验区等地，率先探索建立统一编制、联合报批、共同实施的规划管理体制，试行建设用地指标、收储和出让统一管理机制，探索招商引资、项目审批、市场监管等经济管理权限与行政区范围适度分离。支持在合作园区共同组建平台公司，协作开发建设运营，建立跨行政区财政协同投入机制，允许合作园区内企业自由选择注册地。以市场化为原则、资本为纽带、平台为载体，推动两省市机场、港口、中欧班列、西部陆海新通道等领域企业采取共同出资、互相持股等模式促进资源整合和高效运营。允许能源、电信、医疗等行业有序提供跨行政区服务。探索经济统计分算方式，支持建立互利共赢的地方留存部分税收分享机制，推进税收征管一体化。

　第十章　共同推动城乡融合发展

　　以缩小城乡区域发展差距为目标，推动要素市场化配置，破除体制机制弊端，加快建设国家城乡融合发展试验区，形成工农互促、城乡互补、协调发展、共同繁荣的新型工农城乡关系。

　第一节　推动城乡要素高效配置

　　促进城乡人口有序流动。在重庆主城和成都加快取消对稳定就业居住3年以上农业转移人口等重点群体的落户限制，推动都市圈内实现户籍准入年限同城化累计互认、居住证互通互认，完善居民户籍迁移便利化政策措施。尽快实现公共资源按常住人口规模配置。完善集体经济组织人力资源培育和开发利用机制，探索农业职业经理人培育模式和多种人才引进方式。维护进城落户农民在农村的土地承包权、宅基地使用权、集体收益分配权，研究通过合资、合作、投资入股等方式保障新村民依法享有农村相关权益。健全统一的人力资源市场体系，加快建立衔接协调的劳动力流动政策体系和交流合作机制。
　　深化城乡土地制度改革。探索工业项目标准地出让，探索建立国有土地使用权到期续期制度。深化土地用途转用模式探索，推进城镇低效用地再开发，探索混合产业用地供给和点状供地模式，允许不同产业用地类型依法合理转换。完善建设用地使用权分层管理、统筹开发利用制度，促进地下空间开发。
　　增强城乡建设资金保障。鼓励发展基础设施领域不动产投资信托基金，在防范债务风险前提下，推动城市建设投资稳定增长。发挥中央预算内投资作用，支持引导工商资本在农村投资。依法积极拓宽农业农村抵质押物范围，鼓励创新开发适应农业农村发展、农民需求的金融产品。通过发行地方政府专项债券等支持乡村振兴、农村产业融合发展等领域建设项目。运用大数据、区块链等技术，提高涉农信贷风险管理水平，优化普惠金融发展环境。

　第二节　推动城乡公共资源均衡配置

　　推动城乡一体规划。加快推进“多规合一”，统筹生产、生活、生态、安全需要，建立国土空间规划留白机制和动态调整机制，提高规划适应性。开展城市体检，查找城市规划建设管理存在的风险和问题，探索可持续的城市更新模式，有序推进老旧小区、老旧厂区、老旧街区及城中村改造。强化城市风貌管理，促进建筑物设计更加适用、经济、绿色、美观，推动天际线、街道立面、建筑色彩更加协调，严格控制超高层建筑建设。加强历史文化街区、古镇古村、全国重点文物保护单位等遗产遗迹的整体保护和合理利用，延续城市和乡村文脉，保护传统的山水城格局。严禁违背农民意愿和超越发展阶段撤村并居。
　　推动城乡基础设施一体化。完善级配合理的城乡路网和衔接便利的公交网络。推进城市电力、通信、供水、燃气、污水收集等市政管网升级改造和向乡村延伸，合理建设城市地下综合管廊。开展国家数字乡村试点。大力推进生活垃圾分类处理及再生利用设施建设，全面改善城乡居民卫生环境。加强城乡无障碍设施建设和设施适老化改造。推进城市公共基础设施管护资源、模式和手段逐步向乡村延伸，明确乡村基础设施产权归属，合理确定管护标准和模式，以政府购买服务等方式引入专业化机构管理运行。优化应急避难场所布局，完善抗震、防洪、排涝、消防等安全设施。
　　推动城乡基本公共服务均等化。建立城乡教育联合体和县域医共体。深化义务教育阶段教师“县管校聘”管理改革，鼓励招募优秀退休教师到乡村和基层学校支教讲学，动态调整乡村教师岗位生活补助标准，在职称评审和分配特级教师名额时适当向农村薄弱学校倾斜。加快基层医疗卫生机构标准化建设，提高医护人员专业技术水平，对在农村基层工作的卫生技术人员在职称晋升等方面给予政策倾斜，推动对符合条件的全科医生实行“乡管村用”。把防止返贫摆在重要位置。
　　健全城乡基层治理体系。健全党组织领导的自治、法治、德治相结合的城乡基层治理体系，加强农村新型经济组织和社会组织的党建工作。培育专业化社会组织和社会工作者队伍，调动企业履行社会责任积极性，畅通公众参与城乡治理渠道，推动政府、社会、企业、居民多方共治。推动基层治理重心下沉，完善社区网格化管理体系和便民服务体系，显著提升社区在流动人口服务管理、公共服务提供、社情民意收集等方面的作用。积极运用现代化手段，推动实现智慧化治理。妥善解决村改社区遗留问题。加强交界地带管理联动，建立重大工程项目选址协商制度，充分征求毗邻地区意见。

　第三节　推动城乡产业协同发展

　　依托相应公共资源交易平台，推动农村集体经营性建设用地、承包地经营权、宅基地使用权、集体林权等依法流转和高效配置，盘活农村闲置资源资产。培育高品质特色小镇，着力发展优势主导特色产业。优化提升美丽乡村和各类农业园区，创建一批城乡融合发展典型项目，打造城乡产业协同发展先行区。

　第十一章　强化公共服务共建共享

　　以更好满足人民群众美好生活需要为目标，扩大民生保障覆盖面，提升公共服务质量和水平，不断增强人民群众获得感、幸福感、安全感。

　第一节　推进基本公共服务标准化便利化

　　建立基本公共服务标准体系。实施基本公共服务标准化管理，以标准化促进均等化、普惠化、便利化。联合制定基本公共服务标准，建立标准动态调整机制，合理增加保障项目，稳妥提高保障标准。创新政府公共服务投入机制，鼓励社会力量参与公共服务供给。加大双城经济圈对周边地区支持力度，保障基本公共服务全覆盖。
　　提升基本公共服务便利化水平。共建公共就业综合服务平台，打造“智汇巴蜀”、“才兴川渝”人力资源品牌。重庆市和四川省互设劳务办事机构，推动农民工劳务企业规范化发展。支持探索发展灵活共享就业方式，强化对灵活就业人员的就业服务和权益保障。加快实现双城经济圈社会保险关系无障碍转移接续，推动养老金领取资格核查互认，加快推进全国统一医保信息平台跨省异地就医管理子系统建设，推进跨省市异地就医门急诊医疗直接结算，推进工伤认定和保险待遇政策统一。建设统一的社会保险公共服务平台，推广以社会保障卡为载体的“一卡通”服务管理模式。将常住人口纳入城镇公共租赁住房保障范围，逐步实现住房公积金转移接续和异地贷款信息共享、政策协同。

　第二节　共享教育文化体育资源

　　推动教育合作发展。扩大普惠性幼儿园供给，加大对社会力量开展托育服务的支持力度。鼓励有条件的中小学集团化办学、开展对口帮扶，完善进城务工人员随迁子女就学和在流入地升学考试的政策措施。统筹职业教育布局和专业设置，扩大招生规模，打造一批职业教育基地。建设一批实训基地和国家级创业孵化基地，联手打造“巴蜀工匠”职业技能大赛品牌，打造有区域特色的产教融合行业、企业和院校。组建双城经济圈高校联盟，联手开展世界一流大学和一流学科建设，支持高校向区域性中心城市布局。建设城乡义务教育一体化发展试验区。支持引进境外高水平大学开展中外合作办学，允许外国教育机构、其他组织和个人在自由贸易试验区内单独设立非学制类职业培训机构、学制类职业教育机构，支持建设国际合作教育园区。
　　构建现代公共文化服务体系。构建“书香成渝”全民阅读服务体系，鼓励博物馆、美术馆、文化馆等建立合作联盟，实现公共文化资源共享。建设三星堆国家文物保护利用示范区。推动出版、影视、舞台艺术发展，共同打造“成渝地·巴蜀情”等文化品牌。放宽文化演艺准入，研究建设文化艺术品和文物拍卖中心。建立非物质文化遗产保护协调机制，支持川剧、蜀锦、羌绣、夏布等非物质文化遗产的保护传承发展，研究建设巴蜀非遗文化产业园。
　　共同推进体育事业发展。促进全民健身，推动公共体育场馆、全民健身活动中心、体育公园、社区体育场地等资源设施建设和开放共享，支持公办中小学校和高校的体育场馆、附属设施向社会分时段开放。建立成渝体育产业联盟，支持建设国家级足球竞训基地等专业场地，推动体育项目合作和竞技人才交流培养，协同申办国际国内高水准大型体育赛事。

　第三节　推动公共卫生和医疗养老合作

　　构建强大公共卫生服务体系。增强公共卫生早期监测预警能力。健全重大突发公共卫生事件医疗救治体系，建设省级和市地级重大疫情救治基地、公共卫生综合临床中心。分级推动城市传染病救治体系建设，实现地级市传染病医院全覆盖，加强县级医院感染疾病科和相对独立的传染病区建设。提高公共卫生应急能力，完善联防联控常态机制。加强公共卫生应急物资储备，提升应急物资生产动员能力。
　　优化医疗资源配置。依托四川大学华西医院、重庆医科大学附属医院等优质医疗资源，加快建设国家医学中心。支持共建区域医疗中心和国家临床重点专科群。推进国家老年疾病临床医学研究中心创新基地建设，支持重庆整合有关资源建设国家儿童区域医疗中心，推进四川省儿童医学中心建设。深化中医药创新协作。推动优质医疗资源下沉，支持医联体建设和跨区办医，推动中心城市三甲医院异地设置医疗机构。加强基层医疗卫生服务体系和全科医生队伍建设，构建更加成熟定型的分级诊疗制度。发展在线医疗，建立区域专科联盟和远程医疗协作体系，实现会诊、联网挂号等远程医疗服务。完善二级以上医疗机构医学检验结果互认和双向转诊合作机制。
　　推进养老服务体系共建共享。开展普惠养老城企联动专项行动，发展居家养老、社区养老、机构养老，构建综合连续、覆盖城乡的老年健康服务体系。支持以市场化方式稳妥设立养老产业发展引导基金，制定产业资本和品牌机构进入养老市场指引，支持民营养老机构品牌化、连锁化发展。推动老年人照护需求评估、老年人入住评估等互通互认。鼓励养老设施跨区域共建。统筹医疗卫生和养老服务资源，促进医养融合。推动人口信息互通共享，率先建立人口发展监测分析系统，开展积极应对人口老龄化综合创新试点。

　第四节　健全应急联动机制

　　健全公共安全风险防控标准和规划体系。强化防灾备灾体系和能力建设，完善重大灾害事件预防处理和紧急救援联动机制，加快建设国家西南区域应急救援中心以及物资储备中心，打造2小时应急救援圈，推进防灾减灾救灾一体化。建立健全安全生产责任体系和联动长效机制，有效防范和坚决遏制重特大安全生产事故发生。推广实施公共设施平急两用改造，提升平急转换能力。在跨界毗邻地区，按可达性统筹120、110等服务范围。

　第十二章　推进规划实施

　　加强党对成渝地区双城经济圈建设的领导，明确各级党委和政府职责，细化各项政策措施，建立健全协同实施机制，确保规划纲要主要目标和任务顺利实现。

　第一节　加强党的集中统一领导

　　坚定不移加强党的全面领导，增强“四个意识”，坚定“四个自信”，做到“两个维护”。充分发挥党总揽全局、协调各方的领导核心作用，把党的领导始终贯穿成渝地区双城经济圈建设重大事项决策、重大规划制定调整等各方面全过程。充分发挥党的各级组织在推进成渝地区双城经济圈建设中的领导作用和战斗堡垒作用，激励干部锐意进取、担当作为，组织动员全社会力量落实规划纲要，形成强大合力。

　第二节　强化组织实施

　　重庆市、四川省作为成渝地区双城经济圈建设的责任主体，要明确工作分工，完善工作机制，共同研究制定年度工作计划，落实工作责任，把规划纲要确定的各项任务落到实处。中央有关部门要按照职责分工，加强对规划纲要实施的指导，在规划编制、体制创新、政策制定、项目安排等方面给予积极支持。国家发展改革委要依托城镇化工作暨城乡融合发展工作部际联席会议制度，加强对规划纲要实施的统筹指导，协调解决规划纲要实施中面临的突出问题，督促落实重大事项，适时组织开展评估，及时总结经验做法。重大规划、重大政策、重大项目按程序报批，重大问题及时向党中央、国务院请示报告。

　第三节　完善配套政策体系

　　中央有关部门要加强与重庆市、四川省沟通衔接，负责编制印发实施成渝地区双城经济圈国土空间规划以及多层次轨道交通体系、综合交通发展、西部金融中心建设、科技创新中心建设、生态环境保护、巴蜀文化旅游走廊等规划或实施方案，指导编制践行新发展理念的公园城市示范区、川渝统筹发展示范区、川南渝西融合发展试验区等建设方案，研究出台产业、人才、土地、投资、财政、金融等领域配套政策和综合改革措施。重庆市、四川省编制出台重庆都市圈、成都都市圈发展规划，共同推动形成规划和政策体系，不改变不减弱对三峡库区等周边地区的支持政策。

　第四节　健全合作机制

　　健全推动成渝地区双城经济圈建设重庆四川党政联席会议机制，研究落实重点任务、重大改革、重大项目等，压茬推进各项任务。建立交通、产业、创新、市场、资源环境、公共服务等专项合作机制，分领域策划和推进具体合作事项及项目。培育合作文化，鼓励两省市地方建立合作协同机制。推动与东部地区开展干部人才双向交流、挂职任职。广泛听取社会各界意见和建议，营造全社会共同推动成渝地区双城经济圈建设的良好氛围。

（新华社北京2021年10月20日电）

© 北大法宝：（[www.pkulaw.com](https://www.pkulaw.com/)) 专业提供法律信息、法学知识和法律软件领域各类解决方案。北大法宝为您提供丰富的参考资料，正式引用法规条文时请与标准文本核对。
欢迎查看所有[产品和服务](https://www.pkulaw.com/helps/2.html)。法宝快讯：[如何快速找到您需要的检索结果？](https://www.pkulaw.com/helps/70.html)[法宝](https://www.pkulaw.com/helps/69.html)V6有何新特色？

扫描二维码阅读原文

**原文链接：**<https://www.pkulaw.com/chl/de8a15715863705ebdfb.html>
